# Exchange Transfusion in Neonatal Sepsis: A Narrative Literature Review of Pros and Cons

**DOI:** 10.3390/jcm11051240

**Published:** 2022-02-24

**Authors:** Shigeo Iijima

**Affiliations:** Department of Regional Neonatal-Perinatal Medicine, Hamamatsu University School of Medicine, Hamamatsu 4313192, Japan; siijima@hama-med.ac.jp; Tel.: +81-53-435-2312

**Keywords:** whole blood exchange transfusion, neonatal sepsis, septic shock, adverse event

## Abstract

Neonatal sepsis remains a leading cause of morbidity and mortality worldwide. It is widely considered that exchange transfusion (ET) as an adjunctive treatment for neonatal sepsis has the ability to reduce mortality. This review summarizes the current knowledge regarding the efficacy of ET for neonatal sepsis. In neonatal sepsis, immune responses such as proinflammatory and anti-inflammatory cytokines play an important role in pathogenesis and can lead to septic shock, multiple organ failure, and death. Between the 1970s and 1990s several authors reported that ET was effective in the treatment of neonatal sepsis with sclerema. ET removes bacterial toxins and inflammatory cytokines from the blood by replacing it with fresh and immunologically abundant blood, thereby leading to improvement in tissue perfusion and oxygenation. Moreover, ET with fresh whole blood increases neutrophil count and immunoglobulin levels as well as enhancing neutrophil function. However, there is a lack of clear evidence for the clinical efficacy of ET. In addition, adverse events associated with ET have been reported. Although most complications are transient, ET can lead to life-threatening complications. Therefore, ET can be considered a last resort treatment to rescue neonates with severe sepsis with sclerema and disseminated intravascular coagulation.

## 1. Introduction

Sepsis is one of the most common infectious conditions that occurs during the neonatal period. Although advancements in prenatal screening and intrapartum antibiotic prophylaxis over the past two decades have led to a significant reduction in risk and severity, sepsis remains a major cause of morbidity and mortality in neonates. Globally, an estimated three million neonates develop sepsis every year, and 11–19% of neonatal mortality is related to sepsis [1]. The incidence of neonatal sepsis is inversely associated with gestational age and birth weight. While up to 20% of very low-birthweight infants develop sepsis, the rate in most immature infants may be up to 60% [2], with a mortality rate of up to 71% in neonates weighing <1000 g at the onset of sepsis [3]. Despite the development of new antibiotics and treatment with specific antibiotics, neonates with either severe sepsis along with sclerema (sclerema neonatorum, a unique panniculitis characterized by rapidly progressive hardening of the skin and subcutaneous adipose tissue) or disseminated intravascular coagulation (DIC, a secondary syndrome characterized by excessive systemic activation of coagulation resulting in both hemorrhage and thrombosis) present high mortality rates [4,5]. Moreover, the incidence of neonatal sepsis has increased with the increasing survival of highly-susceptible preterm and/or low-birthweight infants.

A greater understanding of the pathophysiology of sepsis has led to multiple potential therapeutic targets for interventions to improve outcomes. Although routine treatment strategies have remained almost unchanged, to demonstrate enhancement in the host defense mechanisms several types of adjunctive therapies including granulocyte transfusion [6], granulocyte-colony stimulating factors (G-CSF) [7], and intravenous immunoglobulins (IVIG) [8] have been utilized and studied in cases of neonatal septicemia with varying degrees of success. However, these therapies have been ineffective in improving treatment outcomes [9,10]. In such a scenario, clinicians often attempt drastic but unproven and more invasive treatment methods to save rapidly deteriorating neonates with severe sepsis. Exchange transfusion (ET) is one such treatment method; it can rapidly remove circulating bacterial endotoxins and inflammatory cytokines. In the 1970s, case reports noted the effective use of ET in severe neonatal infections along with sclerema [11,12,13]. Thereafter, especially in the 1980s and 1990s, ET was proposed as an adjunct therapy to improve the survival in neonates with severe sepsis [14,15,16,17,18]. However, only a few studies have reported the impact of this treatment (such as reduction in the mortality rate of neonatal sepsis), and reporting is particularly sparse in developing countries [17,18]. Moreover, no definitive recommendations have been provided on the use of ET in neonates with septicemia.

In this narrative review, we summarize the current knowledge regarding neonatal sepsis and ET as a treatment method for sepsis, demonstrate the most significant new findings concerning the efficacy and adverse effects of ET by using diverse data, and introduce alternative therapeutic candidate to ET for the optimal management of neonatal sepsis in order to guide clinicians confronting this life-threatening condition.

## 2. Literature Research Methods

A narrative approach was chosen for this literature review, aiming to provide background information on the postulated pathophysiological mechanisms underlying neonatal sepsis and the history and theoretical efficacy of ET as a treatment method. Moreover, we aimed to provide the main topics with a focus on the challenges posed by therapeutical considerations regarding ET.

The literature research was conducted using the well-established PubMed, Cochran Library, Google Scholar, and ResearchGate databases. In addition, we conducted a search using two major internet search engines Google and Yahoo! Animal models, case series, controlled and uncontrolled studies, and meta-analyses were included, while case reports were excluded; only english-language papers were considered. We did not set a specific time window for the research; however, concerning the adverse effects of ET, we focused on studies conducted in the 2000s. This research used various combinations of the following keywords: “neonate”, “infection”, “sepsis”, “septic shock”, “sclerema”, “disseminated intravascular coagulation”, “pathophysiology”, “adjunctive therapies”, “exchange transfusion”, “adverse effect”, “plasma exchange”, and/or “extracorporeal blood purification.” In the first step, papers were screened by abstract and title. Thereafter, the full text of the selected papers was examined. Papers were excluded if their content was not relevant to the topics of neonatal sepsis, ET, or plasma exchange (PE). Additional publications were identified from the references cited in the initial papers.

## 3. Pathophysiology of Neonatal Sepsis

Neonatal sepsis differs from sepsis in adults, and is defined as a systemic inflammatory response syndrome due to suspected or proven infectious etiology in neonates with implications for epidemiology and pathophysiology [19,20]. The clinical spectrum of sepsis begins when a systemic or localized infection progresses to sepsis. Further clinical deterioration leads to septic shock through the persistence of hypoperfusion or hypotension despite adequate fluid resuscitation and use of vasopressor agents, ultimately leading to multiorgan dysfunction syndrome and possibly death.

**Figure 1:**
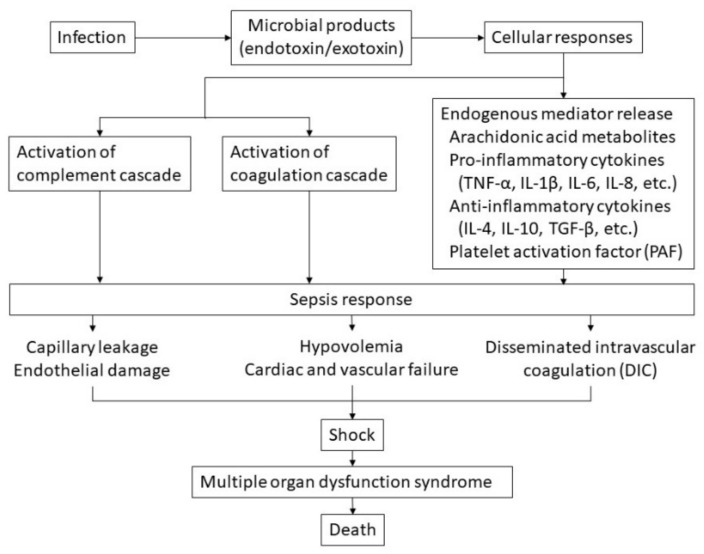
Hypothetical pathophysiology of sepsis. The cascade of sepsis and its influencing factors are shown.

A pathogen in the bloodstream is recognized by the innate immune system and consumed by macrophages and other phagocytes, leading to lysis of the organism [21]. This leads to the release of an endotoxin known as lipopolysaccharide, a component of a gram-negative organism, and an exotoxin known as peptidoglycan, a component of a gram-positive organism. Activation of macrophages stimulates the release of arachidonic acid metabolites such as prostaglandins and proinflammatory cytokines such as tumor necrosis factor α (TNF-α) and interleukin (IL)-1β, IL-6, and IL-8. This in turn leads to the release of anti-inflammatory cytokines, including IL-4, IL-10, and transforming growth factor-β, further activating the complement and coagulation cascades [20]. The complement cascade can activate coagulation and proinflammatory cytokine production and provoke tissue injury. In this way, systemic sepsis becomes a multisystem disorder with varying manifestations, such as hypotension and shock in the cardiovascular system; hemopoietic manifestations may include neutropenia, anemia, and DIC (Figure 1) [19].

Particularly in low-birthweight infants, defense against bacterial infections in neonates may be weakened by such factors as neutrophil deficiency, impairment of chemotaxis of neutrophils and monocytes, complement deficiency, and decreased bactericidal activity of neutrophils [14]. Moreover, the imbalance between proinflammatory and anti-inflammatory cytokines appears to be associated with the severity of sepsis in neonates. Thus, the incomplete development of the host defense system of neonates is largely responsible for the high mortality rate of neonatal sepsis.

## 4. Exchange Transfusion as a Treatment Method for Neonatal Sepsis

ET is a form of blood transfusion in which the patient’s total blood volume is replaced within a few hours. It was introduced in the late 1940s to decrease the mortality of hemolytic disease in newborns and to prevent kernicterus due to hyperbilirubinemia in surviving patients. It is used to remove antibody-coated erythrocytes and products of hemolysis such as bilirubin in various immune or non-immune hemolytic diseases. Subsequently, ET has become one of the most commonly performed neonatal procedures for severe hyperbilirubinemia. Over the decades and coupled with advances in prenatal interventions such as anti-Rh-globulin for Rh-negative mothers and postnatal care such as effective phototherapy, indications for ET have significantly decreased due to the decreased incidence of hemolytic disease [22].

Considering the persistently high case fatality rate associated with neonatal sepsis and septic shock in spite of appropriate antimicrobial and optimal supportive therapy, various adjunctive therapies such as granulocyte transfusion, G-CSF, and IVIG, have been evaluated for the treatment of sepsis and septic shock [7,8]. However, none of these strategies has been demonstrated to reduce the mortality rate in cases of sepsis and septic shock [9,10].

In the 1970s, published case reports described the effective use of ET in severe neonatal sepsis with sclerema [11,13]. In subsequent years, particularly in the 1980s and the 1990s, many clinicians used this procedure as a rescue therapy in neonates with severe sepsis [14,15,16,17,18].

The possible rationales for performing ET using fresh, whole, adult blood include the following:

(i) Elimination of bacteria, bacterial toxins, and circulating inflammatory cytokines; Togari et al. investigated ten neonates who received ET for treatment of septic shock and reported that in six of eight patients with positive endotoxins the endotoxins were completely eliminated by ET. All six patients recovered from shock and survived; however, the two patients who remained endotoxin-positive after ET died [23]. Sugiura et al. reported that in an extremely low-birthweight infant with septic shock due to necrotizing enterocolitis, the elevated serum IL-8 and calprotectin levels decreased after ET and the blood pressure increased and stabilized [24]. Moreover, Chishiki et al. reported that ET saved the life of a neonate with septic shock due to severe Group B streptococcus meningitis, and that the procedure reduced the levels of both proinflammatory and anti-inflammatory cytokines [25].
(ii) Improvement of pulmonary perfusion, ventilation, and tissue oxygenation; Gottuso et al. reported significantly improved pulmonary perfusion and ventilation after ET in low-birth-weight infants with severe respiratory distress [26]. The mechanism for this (as suggested by Delivoria-Papadopoulos et al.) is the improved oxygenation of the tissues following ET with fresh whole blood as a result of either the increase in 2,3 diphosphoglycerate or adult hemoglobin or the removal of vasoactive substances such as prostaglandin F2α [27].
(iii) Correction of the plasma coagulation system; it is thought that ET supplements various coagulation factors, increases the platelet count, and affects DIC to improve coagulation ability [28]. Ugwu et al. reported the case of a neonate with DIC due to severe infection whose coagulation profile was corrected by replacement of consumed coagulation factors using ET and whose clinical condition improved [29].
(iv) Enhancement of immunological defense mechanisms; Sadana et al. demonstrated that serum immunoglobulin (Ig)G, IgA, and IgM levels rose significantly 12–24 h after ET in neonatal sepsis with sclerema [17]. Hall et al. reported significantly increased peripheral neutrophil counts after ET among survivors in neonates with early onset Group B streptococcal septicemia [30]. Furthermore, Mathur et al. reported that leukocyte/neutrophil count increased and granulocyte function improved significantly immediately after ET in septicemic neonates [31]. Suppression of lipid peroxidation [32] and increased blood volume and oxygen supply in the brain [33] have been reported as well.

## 5. Adverse Effects of Exchange Transfusion

ET is not a risk-free procedure, and adverse events associated with ET have been reported even in healthcare settings with advanced clinical care [34,35]. As ET may lead to changes in the internal environment of neonates, adverse events associated with ET may be attributable to fluctuations in blood volume and pressure, changes in acid–base status, alterations in platelet count due to the use of packed red cells that lack platelets and coagulation factors, electrolyte disturbances, and the introduction of infectious agents. The frequency of ET-related adverse events varies in different studies (15–74%) [36,37]. The reasons behind the wide variation in the frequencies of different adverse events include differences in the definition of adverse events, enrollment criteria, and inconsistent follow-up periods after ET. Although the frequency of ET has declined remarkably, the incidence of ET-related morbidity and mortality has not followed suit [38]. Table 1 shows the complications of ET and their incidence reported in studies conducted in the 2000s, although these are all studies on ET for neonatal hyperbilirubinemia [34,35,39,40,41,42,43,44,45,46,47,48]. The most common complications related to ET procedures are thrombocytopenia and hypocalcemia [38,49]. It was found that most adverse events resolve without special treatment [36,40]. However, ET can lead to more severe complications, such as life-threatening bleeding, sepsis, cardiac dysfunction, necrotizing enterocolitis, and even death, apart from transient hypocalcemia, hyperkalemia, bradycardia, and thrombocytopenia. The mortality rate attributable to the ET procedure has been reported to be 0–8% in several previous studies [34,38,39,43,48,50].

Few studies have reported adverse events associated with ET in neonatal sepsis. Pungi et al. reported that serious adverse events were not attributable to the ET procedure in 50 neonates with septic shock [28]. Although approximately half of all patients required platelet transfusion after ET, none of them had bleeding associated with reduction in platelet counts. Moreover, the authors reported that there was no hypocalcemia during or after ET. Aradhya et al. reported that among 41 patients who received ET for sepsis, twelve (29%) developed mild hypothermia, two (5%) had transient bradycardia, two (5%) had hyperkalemia, and two (5%) had hypernatremia, all of whom recovered spontaneously [51]. Verma et al. reported that among seven neonates who required ET for severe sepsis, 14% developed hyperkalemia and 71% developed moderate thrombocytopenia, and all patients recovered spontaneously [52]; however, only one patient developed necrotizing enterocolitis within 12 h of ET. Adverse events of ET in patients with poor clinical conditions such as sepsis may differ in severity from those of ET in patients with hyperbilirubinemia. In their study regarding adverse events related to ET for hyperbilirubinemia Patra et al. observed a high incidence of adverse events, as the enrolled neonates had a clinical condition with a more severe profile [49]. Jackson reported that among all patients who underwent ET for hyperbilirubinemia, the rate of severe complications observed in ill infants (12%) was significantly greater than that observed in healthy infants (1.2%) [38]. Moreover, the author reported that the mortality rate was 2% in the entire group and 8% in the subset classified as ill [38].

## 6. Efficacy of Exchange Transfusion in Managing Neonatal Sepsis

In 1974, Prod’hom et al. proposed ET as an adjunctive therapy for severely septic neonates [11]. Subsequently, various physicians have attempted ET as a treatment modality for severe septicemia and demonstrated its effectiveness in the treatment of neonatal sepsis and septic shock. Xanthou et al. reported that two critically ill neonates with severe infection associated with sclerema were successfully treated with repeated ET [13]. Töllner et al. examined 22 neonates who received ET for sepsis [12]. Both studies demonstrated impressive clinical improvement and decreased mortality following ET. Vein et al. performed ET on ten neonates with refractory sepsis with progressive sclerema [15]. Seven of the ten patients showed immediate improvement and ultimately survived. Dalvi et al. studied 53 neonates with severe or unresponsive sepsis (51 neonates had sclerema) treated with ET and reported an overall survival rate of 77.4% [16]. However, despite the potential benefits of ET evidence-based clinical efficacy is lacking in these studies, as most were anecdotal reports or used small population sizes without comparative controls. To date, only a small number of randomized controlled trials or case-control studies on the efficacy of ET have been published. Table 2 shows prospective and retrospective studies evaluating the efficacy of ET for neonatal sepsis [17,18,28,31,51,52,53,54,55,56,57]. Among the seven prospective studies, two demonstrated that ET significantly reduced mortality. One of the four retrospective studies reported a significant reduction in mortality in the ET group compared to the non-ET group. Moreover, four of the total eleven studies reported significantly increased levels of serum IG and one study showed significantly increased levels of leukocytes and neutrophils. Although most studies have shown clinical benefits, other authors have reported different results. The majority of studies have inadequate sample sizes and follow-up durations, varying inclusion and exclusion criteria, and different or vague septic shock definitions and criteria for ET. Moreover, they do not have mortality rate as their primary objective. Recently, Mathias et al. conducted a meta-analysis in order to evaluate the effect of ET on mortality in neonatal sepsis [58]. The authors demonstrated a mortality benefit (relative risk: 0.72, *p* = 0.01) and a significant increase in immunological parameters (IG and complement levels) (*p* = 0.02) and neutrophil counts (*p* = 0.03) compared with controls in septic neonates who underwent ET. This study suggested that ET can be an adjunctive method for the treatment of neonatal sepsis, although the certainty of the evidence is low.

Regarding blood products used for ET procedure in the present review (Table 2), eight of the total eleven studies and two of the three studies demonstrating significant reduction in mortality in the ET group used fresh whole blood. While ET using reconstituted blood (packed red blood cells and fresh frozen plasma) is expected to achieve elimination of bacterial toxins and inflammatory cytokines, ET using fresh whole blood is expected to improve neutrophil function. However, until recently the emergent availability of fresh whole blood has been limited in most centers. A Japanese survey examined the availability of blood for ET in neonates and reported that the blood products used in 367 ET cases performed during a three-year period (from 2013 to 2016) all used reconstituted blood [59].

**Figure 2:**
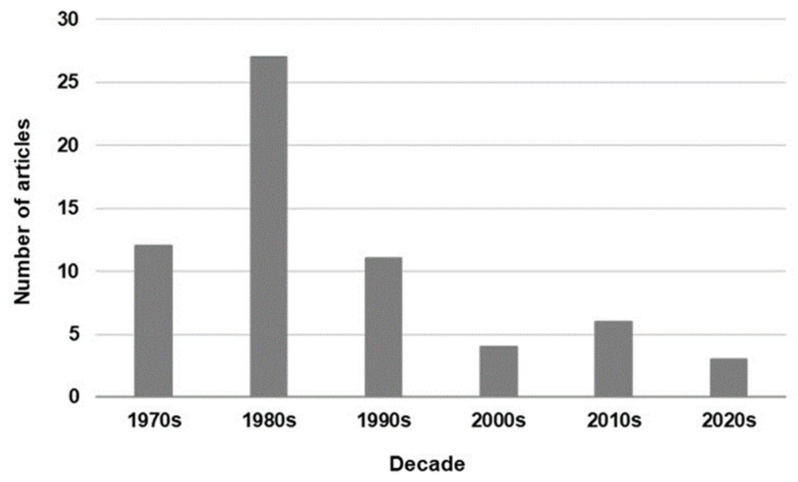
Trend in the number of journal articles on exchange transfusion for neonatal sepsis published per decade.

In this literature review, the first related article found was from the mid-1970s. The number of articles published in the 1980s increased, that in the 1990s decreased, and only a few articles were published in the 2000s (Figure 2). Moreover, in 2016 Cochrane published a review protocol to determine the efficacy of ET in reducing the mortality rate in neonates with septicemia and to ascertain the adverse effects associated with ET; however, the protocol was withdrawn in 2021 because it was out of date [60]. In the latest editions of a textbook on neonatal medicine [61] and in a representative textbook of neonatal infections [62], ET was not suggested as a treatment method for neonatal sepsis.

## 7. Plasma Exchange as an Alternative to Exchange Transfusion

Cytokines play an important role in the pathophysiology of sepsis, even in neonates [20]. Therefore, cytokine elimination therapy may improve the condition of neonates with sepsis. Although ET has been reported to have a cytokine elimination effect [24,25], the direct removal of inflammatory cytokines from whole blood using therapeutic PE is a potential alternative [63]. PE is a well-established therapeutic procedure commonly used in adults; it is based on the removal of plasma from whole blood followed by replacement of that volume with fresh frozen plasma. Evidence suggests that PE can reduce mortality in adult patients with sepsis and septic shock [64]. Several studies have reported that PE in neonates results in improvement of multiple organ failure caused by septicemia [65]. Recently, as the new technology of extracorporeal PE hemoabsorption devices for cytokine reduction (such as Toraymyxin (Toray, Tokyo, Japan) [66] and CytoSorb (CytoSorbents Europe, GmbH, Berlin, Germany) [67,68]) has been introduced, contributing to the achievement of rapid hemodynamic stabilization in neonatal and pediatric patients with refractory septic shock. However, using PE in neonates is considered a challenge due to the relatively large extracorporeal blood volume in relation to the apheresis machines, which are developed for adults. Especially in low-birthweight infants, insufficient blood drainage due to limited vascular access may cause inadequate extracorporeal blood volume to maintain the established flow of the PE system, interruption of the pump, and clotting complications. Moreover, factors such as insertion of large-bore catheters for vascular access and hemodynamic changes due to altered blood volume may make it difficult to use PE as an adjunctive therapy for neonatal sepsis.

## 8. Limitations and Strengths of This Review

This narrative review can be justified on the basis of the diverse data regarding neonatal sepsis and its adjunctive treatment focusing on ET, as well as the needs for this area of interest to be mapped out for the benefit of clinicians engaged in relevant practice.

However, this review has certain limitations. First, the main limitation is the narrative design, which is a source of bias inherent to such studies. The database searches and hand searches referred to the Preferred Reporting Items of Systematic Reviews and Meta-Analyses (PRISMA) guidelines [69]; however, they could not be rigorously followed because this was not a systematic review and meta-analysis. Moreover, study selection was determined by only one researcher. These issues may have resulted in selection bias. However, the author believes that the search was comprehensive with respect to the current state of neonatal sepsis and its therapeutic considerations. Moreover, in order to reduce the impact of publication bias, this review research included grey literature and books in addition to peer-reviewed journals, as these could provide a source of practical experience and important information on neonatal sepsis and ET.

Second, the majority of studies evaluating the efficacy of ET for neonatal sepsis were not recent. The quality of neonatal intensive care and the blood products, devices, and methods used for ET have changed during the last quarter century; evaluations of efficacy and safety made in previous studies may not be acceptable today.

This review did not focus on drawing conclusions as to whether ET is effective for sepsis; instead, the focus has been to comprehensively map the various research results and adverse effects reported to date in order to provide clinicians with information on the respective advantages and disadvantages of this treatment. Moreover, regarding the adverse effects of ET the focus has been on those complications reported in the 2000s, particularly those associated with ET for sepsis. These can be considered strengths of this work.

## 9. Conclusions

Previous studies have shown that ET for neonatal sepsis may have biological plausibility and a probability of benefit. However, the current evidence does neither supports nor refutes the use of ET in the management of severe neonatal sepsis. However, the evidence in question has low certainty, inadequate power, and a moderate-to-high risk of bias. Therefore, adequately powered and well-designed randomized controlled trials are essential for assessing the use of ET for neonatal sepsis. For refractory neonatal sepsis with sclerema and/or DIC, which is likely to be life-threatening, ET can be considered a worthwhile treatment as a last resort. However, ET, which requires the use of fresh whole blood to improve neutrophil function cannot be expected to advance in the future, as the emergent availability of fresh whole blood has until recently been limited in most centers. If a more efficient and safer blood purification method can be developed, ET will not be necessary for neonatal sepsis.

## Figures and Tables

**Table 1:** Adverse events associated with exchange transfusion in neonates and their incidence rates, as reported in studies conducted in the 2000s.

	Chacham et al. [39] 2008–2009 P (*n* = 141)	Malla et al. [40] 2014–2015 P (*n* = 29)	Okulu et al. [41] 2015–2016 P (*n* = 132)	Badiee [42] 2001–2004 R (*n* = 68)	Bülbül et al. [34] 2002–2008 R (*n* = 73)	Yu et al. [43] 2001–2011 R (*n* = 614)	Esfandiarpour et al. [44] 2008–2011 R (*n* = 69)	Chitlangia et al. [35] 2010–2012 R (*n* = 120)	Hakan et al. [45] 2005–2012 R (*n* = 306)	Al-Lawama et al. [46] 2003–2015 R (*n* = 45)	Dey et al. [47] 2016–2019 R (*n* = 41)	Duan et al. [48] 2017–2018 R (*n* = 123)
Death, *n* (%)	9 (6.4)	0 (0)	0 (0)	1 (1.5)	1 (1.4)	0 (0)	0 (0)	5 (4.2)	0 (0)	NA	NA	1 (0.8)
Shock/arrest, *n* (%)	NA	NA	NA	1 (1.5)	NA	1 (0.2)	0 (0)	6 (5.0)	NA	NA	NA	NA
Bradycardia/apnea, *n* (%)	NA	4 (13.8)	NA	1 (1.5)	3 (4.1)	20 (3.3)	1 (1.4)	7 (5.8)	9 (2.9)	2 (4.4)	NA	NA
Respiratory failure, *n* (%)	NA	NA	NA	1 (1.5)	NA	6 (1.0)	NA	11 (9.2)	NA	NA	NA	NA
Sepsis, *n* (%)	7 (5.0)	3 (10.3)	2 (1.5)	NA	1 (1.4)	NA	5 (7.2)	NA	4 (1.3)	NA	8 (19.5)	NA
NEC, *n* (%)	NA	NA	2 (1.5)	1 (1.5)	NA	8 (1.3)	0 (0)	NA	3 (0.9)	NA	NA	2 (1.6)
DIC, *n* (%)	NA	NA	NA	1 (1.5)	NA	1 (0.2)	NA	NA	NA	NA	NA	NA
Metabolic acidosis, *n* (%)	1 (0.7)	NA	NA	NA	NA	191 (31.1)	NA	NA	NA	NA	NA	NA
Hyperglycemia, *n* (%)	NA	15 (51.7)	NA	NA	NA	263 (42.8)	NA	NA	173 (56.5)	7 (15.6)	NA	NA
Hypoglycemia, *n* (%)	NA	1 (3.4)	NA	0 (0)	3 (4.1)	NA	1 (1.4)	NA	3 (0.9)	NA	21 (51.2)	NA
Hypocalcemia, *n* (%)	41 (29)	14 (48.3)	6 (4.5)	2 (2.9)	7 (9.6)	117 (19.1)	0 (0)	118 (98)	69 (22.5)	NA	6 (14.6)	81 (65.9)
Hyperkalemia, *n* (%)	7 (5.0)	2 (6.9)	NA	NA	NA	NA	0 (0)	NA	NA	NA	NA	18 (14.6)
Hypokalemia, *n* (%)	NA	NA	NA	NA	NA	194 (31.6)	NA	NA	NA	NA	NA	35 (28.5)
Hypernatremia, *n* (%)	26 (18)	4 (13.8)	NA	NA	NA	NA	NA	NA	NA	NA	NA	4 (4.1)
Hyponatremia, *n* (%)	8 (5.7)	NA	NA	NA	NA	52 (8.5)	NA	NA	NA	NA	NA	52 (42.3)
Hyperchloremia, *n* (%)	NA	NA	NA	NA	NA	NA	NA	NA	NA	NA	NA	11 (8.9)
Hypochloremia, *n* (%)	NA	NA	NA	NA	NA	NA	NA	NA	NA	NA	NA	27 (22.0)
Hypomagnesemia, *n* (%)	NA	NA	NA	NA	NA	91 (14.8)	NA	NA	NA	2 (4.4)	NA	79 (64.2)
Anemia, *n* (%)	NA	26 (89.7)	NA	NA	2 (2.7)	NA	NA	NA	NA	15 (33.3)	7 (17.1)	NA
Thrombocytopenia, *n* (%)	81 (57.4)	NA	9 (6.8)	4 (5.9)	5 (6.8)	335 (54.6)	0(0)	41 (34)	49 (16)	18 (40)	4 (9.7)	48 (39.0)
Seizures, *n* (%)	4 (2.8)	NA	NA	1 (1.5)	NA	NA	2 (2.9)	NA	NA	NA	NA	NA
Catheter-related, *n* (%)	1 (0.7)	NA	NA	1 (1.5)	NA	NA	NA	NA	23 (7.5)	1 (2.2)	0 (0)	NA

P, prospective study; R, retrospective study, NA, not applicable; NEC, necrotizing enterocolitis; DIC, disseminated intravascular coagulation.

**Table 2:** Previous studies evaluating the efficacy of ET for neonatal sepsis.

Reference	Study Design	Participants	Indication of ET	Blood	Results (Mortality Rate)	Comments
Belohradsky et al., 1979 [53]	R	ET group, 74; no ET group, 132	Preliminary indication scoring system including premature rupture of the membranes, amnionitis, thrombocytopenia, neutropenia, unsuccessful chemotherapy, and positive blood culture	F	ET group, 40/74 (54%); no ET group, 72/132 (55%)	During the last 3 years, only 46% of the ET group died, compared to 82% of the no ET group. The serum levels of IgG, IgM, IgA, and C3 rose after ET.
Bossi et al., 1981 [54]	P	ET group, 22; ST group, 13	Strong clinical suspicion of infection, total leukocyte count < 4000/cum, and at least three of the following five criteria: systolic blood pressure < 40 mmHg, rectal temperature < 36 °C or ≥38 °C base excess ≤ 6.5, platelet count < 100,000/cum, and urine volume < 1 mL/kg/h	F	ET group, 10/22 (45.5%); ST group, 6/13 (46.2%)	Other criteria for indications of ET in severe neonatal septicemia (such as sclerema) should be studied by randomized prospective investigations.
Narayaman et al., 1982 [18]	P	Group I (steroids only), 20; Group II (steroids + blood transfusion), 20; Group III (steroids + ET), 20	Positive blood culture and sclerema and six or more of the following features: lethargy, facial grimace, fever/hypothermia, abdominal distension, intestinal stasis, diarrhea, regurgitation, and respiratory distress	F	Group I, 18/20 (90%); Group II, 14/20 (70%); Group III, 12/20 (60%); *p* < 0.05	In Group III, there were survivors even in the preterm and low-birth-weight infants, whereas in Group I, only tern infants above 2500 g recovered.
Mathur et al., 1993 [31]	P	ET group, 20; Control group, 10	Bacterial sepsis and neutropenia	F	ET group, 7/20 (35%); Control group, 7/10 (70%); *p* = 0.07	Mean total leukocyte count and neutrophil count increased significantly immediately after ET.
Sadana et al., 1997 [17]	P	ET group, 20; Control group, 20	Clinical features of sepsis with sclerema and a positive blood culture	F	ET group, 10/20 (50%); Control group, 19/20 (95%); *p* = 0.0046	The serum IgG, IgA, and IgM levels rose significantly 12–24 h after ET. Greater improvement was observed in survival after ET in more premature infants (28–32 weeks’ gestation).
Gunes et al., 2006 [55]	P	ET group, 33; IVIG group, 33; Control group, 22	Sepsis diagnosed using Töllner’s sepsis score *	F	ET group, 7/33 (21%); IVIG group, 9/33 (27%); Control group, 9/22 (41%); *p* > 0.05	The serum IgM levels rose significantly 12 h after ET and elevated IgM levels persisted for >24 h.
Rajput et al., 2013 [56]	R	ET group, 30; Control group, 30	Septicemic neonates who were critically ill (sclerema)	S	ET group, 14/30 (47%); Control group, 18/30 (60%); *p* = 0.2	Among 38 bacterial sterile neonates (ET, 18; control, 20), mortality was significantly lower in the ET group (5/18 vs. 6/20, *p* = 0.01), whereas among bacterial isolate neonates, mortality was not significantly different (*p* = 0.2).
Aradhya et al., 2016 [51]	P	ET group, 41; ST group, 42	Clinical signs of infection (including sclerema) plus biochemical/radiological/microbiological evidence of infection with objective evidence of organ dysfunction (including metabolic acidosis)	RC	ET group, 14/41 (34%); ST group, 18/42 (42%); *p* = 0.4	Early (within 7 days) mortality (29% vs. 38%, *p* = 0.3) as well as mortality before discharge (34% vs. 45%, *p* = 0.3) showed a trend toward reduction in the ET group. In the ET group, statistically significant improvements in base deficit, IgG, IgM, IgA, and C3 levels were observed.
Pugni et al., 2016 [28]	R	ET group, 50; ScT group, 51	Septic shock defined rigorously in accordance with Goldstein’s and Wynn’s criteria [19]	F	ET group, 36%: ScT group, 51%; *p* = 0.16	The crude OR (95% CI) of death in the ET group was 0.54 (0.24–1.91). In the multivariate logistic regression analysis, after controlling for confounding factors (gestational age, serum lactate, inotropic drugs, and oligo/anuria), ET showed a marked protective effect (OR: 0.21, 95% CI: 0.06–0.71; *p* = 0.01).
Verma et al., 2020 [52]	R	Intervention (ET) group, 7; Control (standard therapy) group, 21	Severe sepsis defined as clinical signs and symptoms consistent with infection, with a screen or culture-positive sepsis, along with evidence of 2 or more organ dysfunction.	RC	ET group, 4/7 (57%); Control group, 15/21 (71%); *p* = 0.004	There was a significant reduction both in the incidence of refractory shock (71% vs. 75%; *p* = 0.01).
Chillal et al., 2021 [57]	P	ET group, 43; ScT group, 43	Clinical picture (sclerema) and total leucocyte count < 5000/cum, absolute neutrophil count < 1800/cum and CRP > 1 mg/dL	F	ET group, 12/43 (28%); ScT group, 13/43 (30%); *p* > 0.05	There was a significant reduction in the mean duration of hospital stay and antibiotic use in the ET group.

* Töllner’s sepsis score consists of clinical (skin color, body temperature, muscle tonus, breath rate, abdominal distension, imperfect microcirculation, and risk factors) and laboratory (leukocyte and thrombocyte counts, C-reactive protein, RP, and immature/total neutrophil ratio) parameters. Patients who have >10 points are determined as having sepsis. ET, exchange transfusion; R, retrospective study; P, prospective study; F, fresh whole blood; ST, standard therapy; S, stored whole blood; RC, reconstituted blood (packed red blood cells and fresh frozen plasma); ScT, standard care therapy; OR, odds ratio; CI, confidence interval.

## Data Availability

Not applicable.
